# Mapping genes with longitudinal phenotypes via Bayesian posterior probabilities

**DOI:** 10.1186/1753-6561-8-S1-S81

**Published:** 2014-06-17

**Authors:** Anthony Musolf, Alejandro Q Nato, Douglas Londono, Lisheng Zhou, Tara C Matise, Derek Gordon

**Affiliations:** 1 Department of Genetics, Rutgers University, 145 Bevier Road, Piscataway, NJ 08854, USA

## Abstract

Most association studies focus on disease risk, with less attention paid to disease progression or severity. These phenotypes require longitudinal data. This paper presents a new method for analyzing longitudinal data to map genes in both population-based and family-based studies. Using simulated systolic blood pressure measurements obtained from Genetic Analysis Workshop 18, we cluster the phenotype data into trajectory subgroups. We then use the Bayesian posterior probability of being in the high subgroup as a quantitative trait in an association analysis with genotype data. This method maintains high power (>80%) in locating genes known to affect the simulated phenotype for most specified significance levels (α). We believe that this method can be useful to aid in the discovery of genes that affect severity or progression of disease.

## Background

Current association studies focus primarily on disease susceptibility, searching for correlations between genetic variants and disease phenotypes. Studies looking for association with the severity or progression of a disease have been less frequent. This may be partially because these phenotypes require multiple data points across time, which require more time and money to collect properly. When longitudinal data are collected, however, studies show they can be used to accurately map genes. One example involves the progression of spine curvature in scoliosis [1].

Historically, biological studies have been restricted in their use of longitudinal phenotypes. Breakthroughs in the field of growth mixture models, such as random effects modeling [2], have allowed geneticists to begin to analyze longitudinal data more effectively. The result has been a substantial increase in the number of new studies that use these growth mixture models to detect genes responsible for growth or progression trajectories [3]. One of the particular benefits of growth mixture models is their ability to classify heterogeneous data into distinct trajectory subgroups or to identify smaller groups within a larger phenotype group. The probability of an individual belonging to a particular subgroup, called the Bayesian posterior probability (BPP), can be calculated and used in association analyses. This is evidenced by Kerner and Muthen [4], who classified patients into phenotype subgroups and performed association tests on subgroup membership and single-nucleotide polymorphisms (SNPs).

In this study, we present a novel method of mapping genes using longitudinal data that were obtained from Genetic Analysis Workshop 18 (GAW18). Our disease of interest is hypertension, and we use the simulated systolic blood pressure (SBP) values as our phenotype. We performed a population-based study using unrelated individuals and a family-based study using extended pedigrees. Our approach involved assigning individuals into trajectory subgroups and testing for association with SNPs using the BPP of being in the clinically relevant subgroup as a phenotype. Because hypertension is the disease of interest in this study, we define the subgroup with the highest SBP as the clinically relevant group. Power for given significance levels was then calculated on genes determined by GAW18 to affect the simulated SBP phenotype.

## Methods

### Replicate set creation and analysis of longitudinal data

Both phenotype and genotype data were provided by GAW18. We used the simulated SBP values as our phenotype, which contained SBP values at 3 time points for 850 individuals. GAW18 created 200 phenotype files (to serve as replicates), each containing the same 850 individuals with different SBP values at each time point. The analyses were performed on 2 studies, one was population-based and the other family-based. Genotype data included both sequenced and imputed data free of mendelian errors. The family-based study used related individuals from 20 extended pedigrees. The population-based study used 157 individuals extracted from the pedigrees determined to be genetically unrelated by GAW18. Three time-varying covariates (age, hypertension medication, smoking status) and 1 time-independent covariate (sex) were used. We performed 2 full analyses on each study, 1 with covariates and 1 without covariates. Thus we had 4 discrete analyses: population-based with covariates, population-based without covariates, family-based with covariates, and family-based without covariates. For each of the studies, we selected a set of 3 replicates by sampling without replacement from the pool of 200 simulated SBP phenotypes created by GAW18. The 3 replicates in a set represent 1 discovery data set and 2 confirmatory data sets. This was repeated 100 times, creating a total of 100 discovery sets and 200 confirmatory sets.

The replicates were then analyzed via SAS PROC TRAJ, which uses mixture modeling to assign longitudinal data into subgroups [5]. Each replicate was evaluated using 6 different models (*k*). Each model generated a different number of subgroup trajectories, where *k *= 1,...,6. Thus, the 1-subgroup model generated a single group while the 6-subgroup model created 6 subgroups from the data. SAS also allows for the specification of the polynomial order of each subgroup trajectory. The initial models used cubic polynomials for all subgroups. Outputs of the initial runs provided a *p *value for each polynomial coefficient. The order of the polynomials in each subgroup was adjusted to that of the highest order polynomials that were still significant at the 5% level in the first run. The analyses were repeated for a second and final run. For the analyses using covariates, the covariates were introduced into the models at this point. We then determined the optimum model (ie, the correct number of subgroups) to be the model with the highest Bayesian information criterion (BIC).

With the optimum model selected, we obtained the BPP that an individual belongs to a particular subgroup. For this study, we determined that the clinically relevant group was the subgroup with the highest value at the last time point predicted by the corresponding polynomial. This correlates with individuals with the most severe hypertension. Thus, we define the highest subgroup as the subgroup with the highest SBP at the final time point. The BPP of each individual belonging to this subgroup was used in the association analyses. We note that by choosing the clinically relevant group based on the highest fitted value at the final time point we always get the group that contains individuals with the highest SBP values, regardless of the total number of subgroups determined by the BIC. We also note that clinical relevance is a function of the disease of interest and that the method can be modified to look at any subgroup.

### Association analyses and power calculations

We performed association analyses using the BPPs as a quantitative trait. The population-based data were analyzed via PLINK's Wald test (assoc command) [6]. We used the false discovery rate as implemented in PLINK to determine overall significance. Association was calculated for the family-based analyses through transmission disequilibrium test (TDT). Two distinct programs were used for TDT calculations. One was PLINK's QFAM procedure, which uses linear regression to fit genotypes to phenotypes and corrects for family structure via permutation for quantitative phenotypes [6]. The second was TDT-HET, an expanded TDT statistic that incorporates locus heterogeneity in families [7]. These 2 programs use permutation to correct for multiple testing. Because TDT-HET requires dichotomous phenotypes, the BPPs were converted to binary for this particular program. This gave us the opportunity to see whether power was lost in the conversion of quantitative phenotypes to dichotomous phenotypes. We converted the estimated posterior probabilities that were above 0.5 to 1 and those below 0.5 to 0 because our BPPs had a bimodal distribution regardless of the total number of subgroups estimated. The composition of the fast group remained the same regardless of whether the BPPs were dichotomized or not. Time constraints caused by the permutation tests necessitated that the family analyses be run only on the region of interest. For the population study, analyses were run on the region of interest and several surrounding regions on the same chromosome.

**Figure 1:**
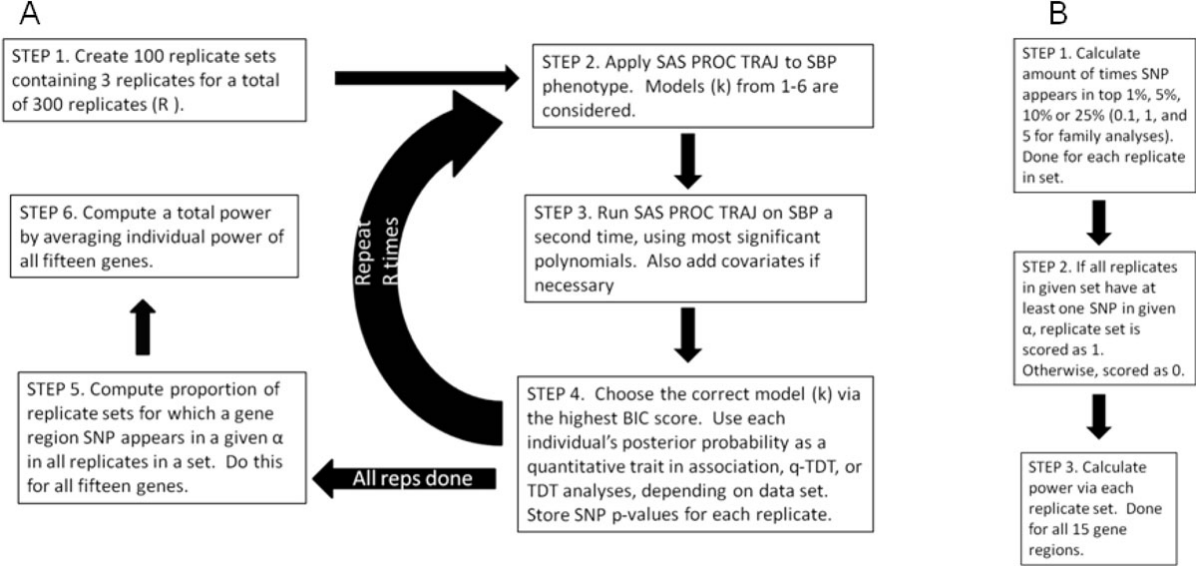
**Flowchart of overall method and power calculation**. A. Flowchart detailing the overall method. B. Flowchart detailing the calculation of power.

Power calculations were then performed on the top 15 genes affecting the simulated SBP phenotype. Two types of power were calculated: per-gene power and total power. Per gene power was calculated for each of the 15 gene regions on a binary, or YES/NO, scale. To constitute a YES, all 3 replicates in a replicate set needed at least 1 marker within a given gene with a *p *value in the top x% (population-based) or below a given threshold, α (family-based). All other scenarios were considered NO. For the population-based data, x% was defined as the top 1%, 5%, and 10%. For the family data, α was defined as SNPs with *p *values at or below 0.1%, 1%, and 5%. We also estimated total power, which was defined as the average of the per-gene power and is intended as a cumulative assessment of the method. T tests were performed at each α level between the noncovariate and covariate analyses to determine whether the addition of covariates was significant. We also performed T tests between corresponding PLINK and TDT-HET to determine whether mean power (per gene and total) of one method was significantly higher than the other. In addition to the gene regions, we applied our method to 3 "null" regions of varying size. A null region was defined as a region not containing any of the simulated functional loci associated with SBP and was used to estimate our method's type I error rate. Figure 1 details the steps involved in our method.

## Results

### Model analysis

For the population-based studies without covariates, the average number of subgroups (*k*) ranged from 3 to 6, mean: 4.78, median: 5, mode: 4. The fast subgroup had an average proportion of 8.89% individuals. When covariates were added to the study, the range shifted from 2 to 5, mean: 3.05, median and mode: 3. The average proportion of individuals increased significantly, to 40.52% (see Discussion). We saw a similar trend in the family studies. Without covariates, *k *ranged from 3 to 5 with mean: 4.22, median and mode: 4. The average proportion of individuals in the fast group was 8.98%. When covariates were included, the range expanded from 2 to 5 with mean: 4.18, median and mode: 4. The average proportion of individuals in the fast group increased to 36.61%. Thus, in both cases, covariates significantly increased the number of individuals within the fast group.

The BPPs in both the population-based and family-based studies had a bimodal distribution. Because our association analysis is regression based, we performed a Box-Cox transformation to normalize the data. We found there was no statistical difference between results using the original data or the transformed data.

### Total power and per-gene power

For the population-based study, we observed total power above 80% at 5% and 10% significance levels. Although power was slightly higher for the covariate analysis, mean power differences were not statistically significant. Observed total power was less than 50% for both data sets at the 1% level. The null regions maintained proper type I error levels. For the family-based study, greater than 80% power was observed using PLINK regardless of covariate inclusion. Total power was less than 50% at both α = 0.1% and α = 1%. Inclusion of covariates did not significant change mean total power at any α level. TDT-HET produced similar results, although slightly higher, than PLINK. T tests comparing TDT-HET and PLINK were significant at α = 0.1% but not at α = 1% or α = 5%. The null regions maintained proper type I error levels.

**Figure 2:**
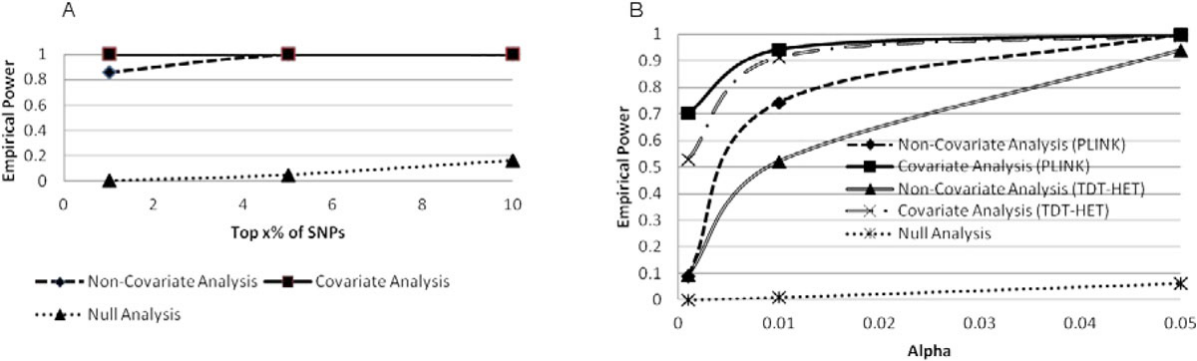
**Per gene power graphs for *MAP4.*** A. Graph of power using population-based analyses. B. Graph of power using family-based analyses.

We also calculated power for each of the top 15 genes affecting SBP (per-gene power). The population-based analyses showed that 12 and 11 genes had greater than 80% power at the 5% and 10% levels, respectively. Only a single gene had greater than 80% power at the 1% levels. We note that this gene was *MAP4*, the top gene affecting the SBP phenotype, accounting for more than 6% of the total variance. Covariates significantly increased power in 5 genes, including *MAP4 *and *NFR1*. Figure 2A shows the results for *MAP4*, along with the results of a null region of comparable size. Per-gene power was also calculated for the family-based analyses. Both PLINK and TDT-HET produced results with 11 of the 15 genes having power above 85% at α = 5% for the noncovariate analyses. Adding covariates increased power to above 90% for 4 genes, including *MAP4*. No statistical difference using T tests was observed between the TDT-HET results and the PLINK results. Figure 2B shows the family results.

## Discussion

Results of our analyses suggest that our method of grouping longitudinal phenotypes into subgroups and using the BPP as a quantitative trait is a robust method for finding association with SNPs in gene regions. We were able to identify genes affecting the SBP phenotype with high power in both family-based and population-based studies. We also observed high per-gene power when using covariates. Our phenotype of interest was hypertension. For this reason, we used the highest group as our clinically relevant group. However, this choice is flexible and any subgroup could be used as clinically relevant. For example, researchers involved with scoliosis might also be interested in the fastest progression group. If a phenotype like renal failure was being investigated, though, researchers might be interested in the fastest decreasing renal function group.

One interesting finding is the effect of covariates on the correct number of subgroups. Covariate inclusion increased the proportion of individuals in the fast group in both studies. Mathematically, this occurred because the covariate analyses tended to have more 2 and 3 subgroup models selected as compared with the noncovariate analyses that identified a larger number of subgroups. That could mean some of the subgroups in the noncovariate analyses are not distinct subgroups, but covariates helped to identify and collapse them.

Another interesting finding was that no power was lost in the conversion of quantitative traits to dichotomous traits for TDT-HET. In fact, power was often gained. We believe this is a result of the bimodal nature of the BPP.

We note that our power at 1% level was less than 50%. We believe that this was caused by our low sample size, given that the effect size of some of the associated genes was known to be small for these data. This is evident in *MAP4*, which accounted for 6% of the total SBP variance. We estimated 80% power using our method even at the 1% level.

Finally, we wanted to compare the performance of our method to methods proven to identify SNP signals associated with longitudinal phenotypes. We chose to compare to the functional genome-wide association studies (fGWAS) software [8]. We found that the results between our method and fGWAS were statistically identical (results not shown). However, we note that it was only an exploratory study; the scope of this study was not a full model comparison.

## Conclusions

Our method for analyzing longitudinal data produced high power for identification of association between BPP of group membership and SNPs in genes known to affect SBP. The method's power was high (greater than 80%) in multiple scenarios, including different genotype data and sample size. The flexible nature of this approach allows it to be used in a variety of tests, including exploratory analysis of the entire genome or confirmation analysis on a given region. Significant increases in power with covariate inclusion also show our method's ability to detect interactions between genetic and environmental factors. This provides the prospective researcher the potential to effectively analyze environmental covariates during an association study with longitudinal data. Based on our power results, we believe this method can be an effective and efficient approach to analyzing longitudinal data from a variety of different data sets and study designs.

## Competing interests

The authors declare that they have no competing interests.

## Authors' contributions

DG, DL, and TCM designed the overall study and supervised the analyses. AM performed all analyses except TDT-HET and drafted the manuscript. AQN performed the TDT-HET analyses. LZ performed quality control and data cleaning.
